# Predictors of concussion management pathway adherence in New Zealand community rugby: A prospective cohort study

**DOI:** 10.1016/j.jsampl.2026.100161

**Published:** 2026-10-05

**Authors:** Danielle M. Salmon, Janelle Romanchuk, Marelise Badenhorst, Anja Zoellner, James Brown, Zachary Yukio Kerr, Chris Whatman, Johna Register-Mihalik, Gisela Sole, Simon Walters

**Affiliations:** a New Zealand Rugby, PO Box 2172, Wellington, 6140, New Zealand; b International Rugby Players Association, Dublin, Ireland; c Sport Injury Prevention Research Centre, Faculty of Kinesiology, University of Calgary, Alberta, Canada; d School of Physical Education, Sport and Exercises Sciences, University of Otago, Dunedin, New Zealand; e Sport Research Institute New Zealand, School of Sport, Exercise and Health, Auckland University of Technology, New Zealand; f Carnegie Applied Rugby Research (CARR) Centre, Carnegie School of Sport, Leeds Beckett University, Leeds, UK; g Institute of Sport and Exercise Medicine (ISEM), Department of Exercise, Sport and Lifestyle Medicine, Faculty of Medicine and Health Sciences, Stellenbosch University, Stellenbosch, South Africa; h Division of Physiological Sciences, Department of Human Biology, Faculty of Health Sciences, the University of Cape Town and the Sports Science Institute of South Africa, Cape Town, South Africa; i Matthew Gfeller Sport-Related Traumatic Brain Injury Research Center, Department of Exercise and Sport Science, University of North Carolina at Chapel Hill, Chapel Hill, USA; j Department of Exercise and Sport Science, University of North Carolina at Chapel Hill, Chapel Hill, USA; k Centre of Health, Activity, Rehabilitation Research, School of Physiotherapy, University of Otago, Box 56, Dunedin, 9054, New Zealand

**Keywords:** Rugby, Concussion, Injury, Community, Healthcare

## Abstract

**Background:**

There is a need for improved operationalisation of concussion guidelines in community sport. The inclusion of technology may offer a viable solution. This study aimed to evaluate if adherence to a concussion management pathway (CMP) facilitated through the use of technology, improved compliance to concussion guidelines.

**Methods:**

This prospective observational study (2020–2022) included 5879 players across four unions in NZ. Healthcare touchpoints, demographics, baseline completion, physiotherapist support, and adherence to stand-down periods (SDP) were captured. Generalised linear mixed models examined factors associated with ideal pathway adherence (logged suspected concussion (SC) → system GP diagnosis → system GP medical clearance) and appropriate SDP.

**Results:**

Across three seasons, 639 SC occurred (n = 550 males, n = 89 females), of which 631 were logged. Of these logged SCs, 82.6% were medically confirmed. The overall SC rate was 16.6 per 1000 match hours. Approximately half (49.9%) adhered to the pathway. The strongest predictor of pathway adherence was completion of a pre-season baseline (OR = 1.90). Physiotherapist support was not significant, but was strongly associated with adherence in one union (OR = 6.15). Pasifika/Other players had lower odds of pathway adherence than NZ European (OR = 0.60). Pathway followers were more likely to adhere to the SDP (79.3% v 45.6%) and RTP that season (85.0% v 57.8%).

**Conclusion:**

Adherence to the pathway was associated with greater compliance to the recommended SDP and increased likelihood of RTP that season. Pre-season baseline was the most consistent predictor of pathway adherence. Integrating digital systems, pre-season assessment and clinical support may strengthen implementation of community concussion guidelines.

**Key points:**

• A technology-enabled concussion management pathway supported adherence to community concussion guidelines by linking pitch-side recognition with medical diagnosis and clearance, with 49.9% of players completing the ideal pathway across three seasons
• Players who followed the ideal pathway were more likely to observe the recommended stand-down period and return to play safely.
• Completion of a pre-season baseline assessment was the strongest predictor of ideal pathway adherence, where completing a baseline assessment improved the odds that a player would follow the ideal pathway by 90%.
• The value of team physiotherapist presence was context-dependent: not significant in the primary model overall, but strongly associated with adherence in one provincial union where players access general practitioners through the primary care setting, highlighting the importance of local healthcare infrastructure.
• Digital capture of concussion care touchpoints enhanced surveillance beyond incidence reporting, enabling monitoring of real-world implementation and facilitators of guideline adherence at the community level.

## 1 Introduction

Concussion can result in cognitive, neurological, and functional impairments that significantly affect daily activities, work, education, and overall quality of life [1]. At the community level, World Rugby has established the *Recognise and Remove* guidelines, which require any player who sustains a suspected concussion be immediately removed from play, medically assessed, and medically cleared before returning [2]. Players diagnosed with a concussion must observe a mandatory, minimum 21-day stand-down period (SDP) [3]. However, a key challenge in community rugby is implementing these guidelines consistently in practice.

Implementing concussion management guidelines consistently in community sport remains challenging. Limited pitch-side medical professionals means that recognition of suspected concussion often falls to coaches or team managers, many of whom have minimal formal concussion training [4,5]. Compounding the pitch-side identification, player non-disclosure further complicates concussion identification and subsequent management [[6], [7], [8]]. For example, a study by Travis et al. (2021) examining concussion safeguarding in British American Football revealed inadequate medical provisions across teams, resulting in inconsistent diagnosis and management of return to play (RTP), and concluded that guidelines alone were insufficient to ensure safe sporting environments [9].

Challenges also extend beyond the field to the medical management of concussions within primary care. A pilot project conducted by New Zealand Rugby (NZR) in 2018 asked general practitioners (GPs) to use the SCAT5 for concussion diagnosis [10]. Findings revealed that most GPs were unfamiliar with best-practice return to play (RTP) guidelines, had limited experience using the SCAT5, lacked sufficient consultation time, and saw relatively few (1 or2) concussions per month [10]. Similar findings have been reported from a study in the United States and Canada [11] and from Ireland, where only 12% of GPs had received formal concussion training [12]. A recent scoping review in the UK identified an urgent need for a comprehensive, time-efficient diagnostic tool that integrates seamlessly into primary care systems and supports clinicians in the diagnosis, triage, and management of concussion [13].

Recognising that community guidelines alone were insufficient to support consistent concussion management [9], NZR developed the *Concussion Management Pathway* (CMP), supported by a digital App. The pathway was informed by the structured approach used within World Rugby's professional Head Injury Assessment protocol but adapted to reflect the realities of community rugby, where access to medical professionals at the time of injury is limited. The CMP aimed to support coaches, team managers, physiotherapists, and players in recognising and managing suspected concussion and to provide clear guidance on appropriate post-injury steps before GP diagnosis assessment. Although the associated NZR App facilitated pitch-side management, it did not directly assist GPs in diagnosing or clearing players. To address this, NZR convened an expert panel of GPs, sports medicine specialists, and representatives from the College of GPs to develop the *NZR Concussion Assessment Pathway* (NZRCAP), based on the SCAT framework [14]. A secure, web-based GP portal was created to integrate with the NZR App, providing an accessible clinical decision-support tool that allowed GPs to view players' baseline NZR Concussion Assessment Tool (NZRCAT) scores and record diagnostic findings [10,14]. The CMP framework in Fig. 1 outlines the entire pathway from injury occurrence to Return to Play (RTP). The ideal pathway was defined as the following: a team lead (coach, physiotherapist, or team manager) logging a suspected concussion; the player attending both a diagnostic and medical clearance assessment with a general practitioner (GP) who uses the GP Portal.

**Fig. 1:**
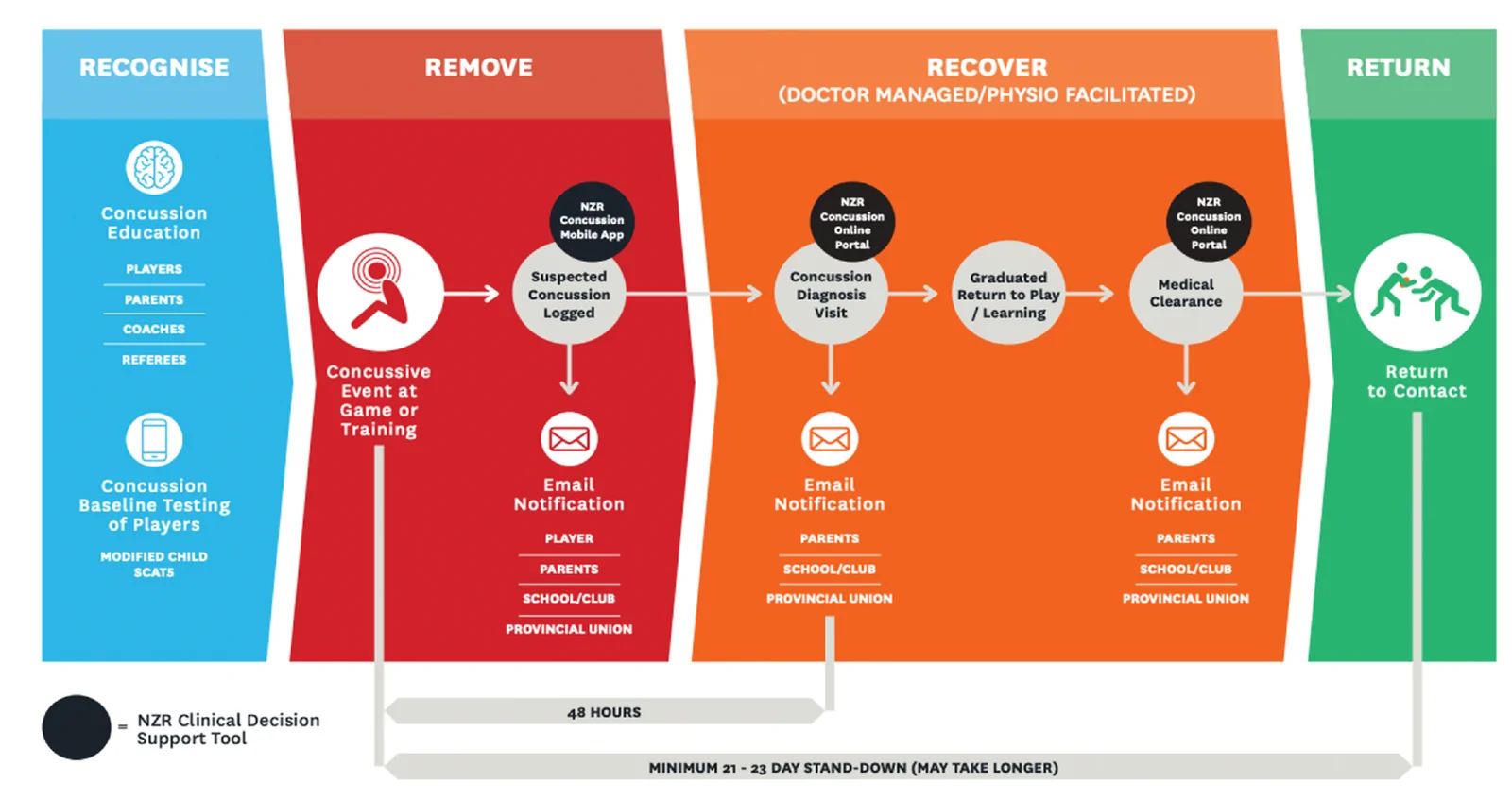
Infographic developed by NZR to educate community stakeholders on the concussion management pathway.

The aim of this study was to evaluate adherence to an ideal concussion management pathway, facilitated through the use of an NZR App and GP Portal. To explore what factors contributed to improved adherence to the ideal pathway the effect of gender, ethnicity, grade, presence of team physiotherapist and completion of a baseline assessment were examined. In addition to pathway adherence, compliance with the appropriate SDP was examined to determine alignment with NZR guidelines in effect at the time of the study (21 days for players aged ≥19 years and 23 days for players aged <19 years) and to identify those who returned early.

## 2 Methods

### 2.1 Study design

This study employed a prospective observational cohort study to examine concussion management adherence across four Provincial Unions in New Zealand (NZ). These Provincial Unions represented diverse cultural, socioeconomic, and geographical locations. This study was conducted and reported in accordance with the STROBE checklist for observational studies (Supplementary Table S1).

### 2.2 Recruitment and consent

Administrators in each Provincial Union provided approval for their region to be included in the study. Upon approval, the research team approached administrators of schools/clubs to explain the study and seek interest in participating. Where teams/clubs were interested, the researchers attended a pre-season training to brief the players. Players were invited to participate and requested to provide individual consent. Guardian consent was also obtained for those players under the age of 16. Ethical approval was obtained from the University of Otago Human Ethics Committee (approval 18/087).

### 2.3 The concussion management pathway

The CMP and the technology that support this process were model on the Head Injury Assessment process in the elite game but collaboratively adapted with community stakeholders over the course of the study. The App guided the management of players through each step in their suspected concussion journey, and the GP Portal provided a clear, structured pathway to assist GPs with their clinical assessment for both diagnosis and medical clearance of a concussion. Team leads used the App to baseline test players and log suspected concussion. The App was also used as a resource to support team leads' recognition of red flags and to provide information to give players during the first 48 h before their medical assessment. The GP Portal was designed in collaboration with experts and community GPs [10]. The purpose of which was to provide an easy to use tool that supported GPs through the NZRCAT where they could see the player's baseline scores, highlights the physical and neurological exams recommended in SCAT5, and contain NZR's RTP guidance that the GP could reference. At the end of this assessment GP's were asked to make a diagnosis or medical clearance depending upon the assessment based on the sport concussion 5th consensus meeting definition of a sport related concussion.

The CMP pathway started during the pre-season, when players were encouraged to complete NZRCAT baseline assessment [14], conducted on the NZR App. During the season, team leads (team manager/coach/physiotherapist) logged suspected concussion that occurred during games and/or training in the NZR App. When logging the suspected concussion in the NZ App, if no red flags were reported, team leads were asked to advise the player to see a GP within 48 h. When the suspected concussion was logged in the App, automated emails were sent to the player, parent/caregiver, school/club and the Provincial Unionnotifying these stakeholders the player had been removed. The emails to the player and parent/caregivers contained a unique code that when provided to the GP enabled them to access their baseline assessment on the GP Portal. For the diagnosis and medical assessments players were able to see their own GP or CMP affiliated GPs who had GP Portal access (referred to as System GPs). To access the GP Portal, GPs had to be set up in the system and go through a training process on how to use the Portal and the recommended assessments. When an assessment was completed on the GP Portal at the end the GP would be asked based on the results of the repeat NZRCAT, their physical exam and their clinical judgement to make decision regarding the diagnosis or medical clearance of the player [14]. For those players who attended a diagnosis or medical clearance assessment with their own GP, the outcomes and timings of these were obtained by a research assistant via phone call or in-person self-report follow ups with the player after the suspected concussion was logged in the NZR App. The health care touchpoints for the CMP are outlined in Fig. 2.

**Fig. 2:**
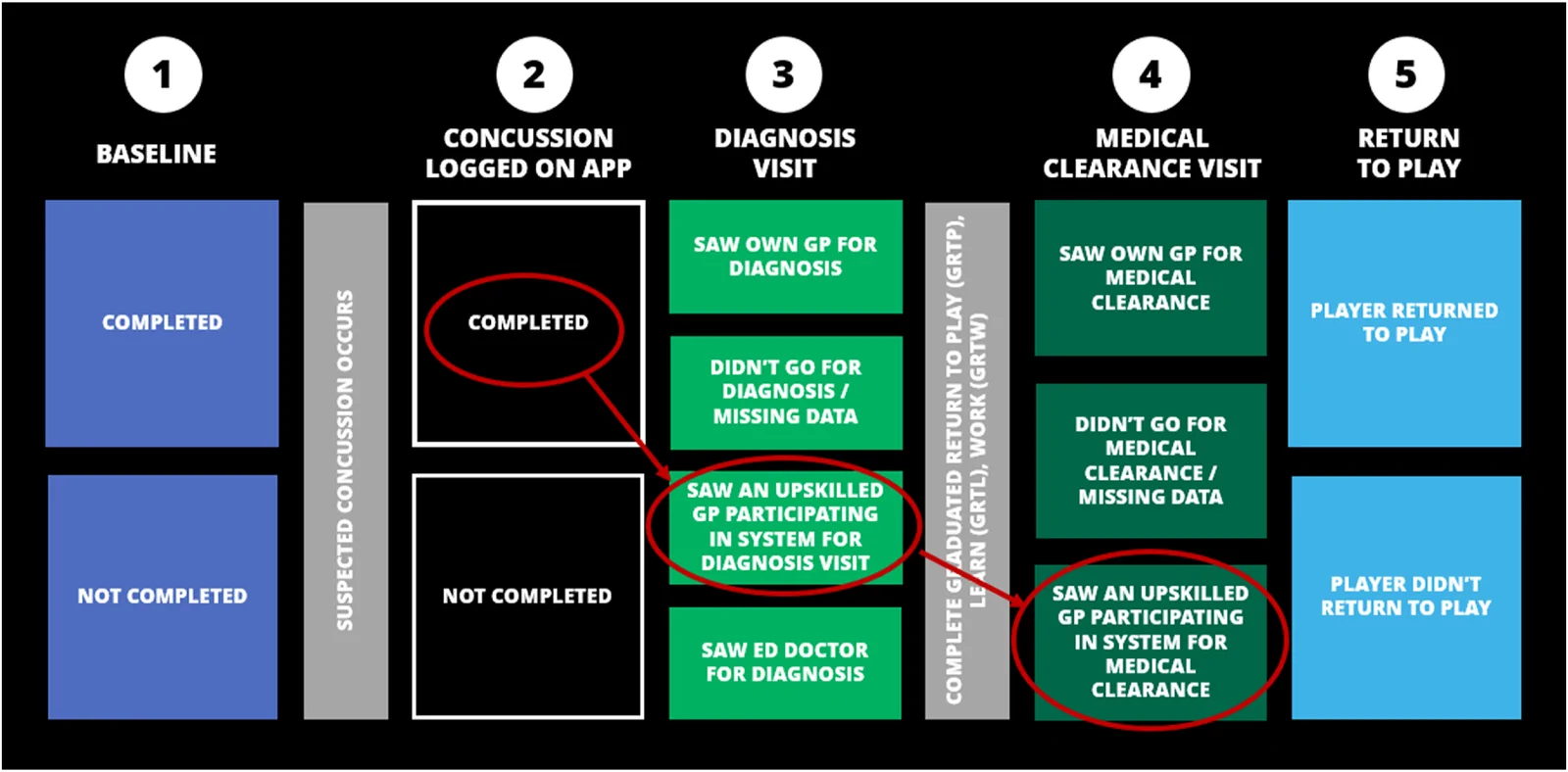
The healthcare touchpoints for the NZR Concussion Management Pathway from the baseline NRZCAT being completed to the player returning to play. Each healthcare touchpoint was coded into the corresponding pathways, then grouped into either ‘ideal’ or ‘alternative’. The ‘ideal’ pathway outlined in red required: Suspected concussion logged in App → GP Diagnosis (System GP) → GP Medical Clearance (System GP).

To track adherence to the ideal pathway for each concussion the healthcare touchpoints were coded and grouped into either ‘ideal’ or ‘alternative’ pathways. The ideal pathway required: that the suspected concussion logged in the App → player attended a GP Diagnosis (System GP) → player attended a GP Medical Clearance (System GP). All other pathways were coded as ‘alternative’. As the research team was notified when a suspected concussion was logged they were able to follow up with players and team leads in real time to track each player's health care touchpoints (system GP, own GP or missing). Post season, research assistants followed up with teams to ensure all injury data was captured and this information was cross checked against the assessment log in the NZR App and GP Portal.

### 2.4 Participants

Four Provincial Unions participated in the study. Provincial Unions 1–3 were involved across all three seasons (2020–2022), while Provincial Union 4 joined in the 2022 season only. Players in Provincial Unions 2–4 accessed GPs through a primary care setting. All players in the study cohort could access GPs through the primary care setting; however, players based in the primary metropolitan area in Provincial Union 1 could also access GPs through an acute primary care concussion clinic. All registered players in consenting high school and club teams were eligible to participate. Over the study period 306 teams participated, representing an eligibility pool of 8537 players (2020: n = 2033; 2021: n = 2691; 2022: n = 3813). A total of 5879 players consented (2020: n = 1530, 2021: n = 1967, 2022; n = 2382).

### 2.5 Data processing

All assessments completed in the App and GP Portal were linked and exported, data were cleaned, and duplicates removed. Assessments where players had seen GPs outside the system were added to the data set. Post-season the teams were contacted and training and match exposure was captured, and any suspected concussions that were not logged in the App were recorded.

### 2.6 Data analysis

Data were analysed using R (Version 4.5). Descriptive statistics were presented for demographics, baseline completion, presence of a team physiotherapist, RTP outcomes, and SDP adherence. Two measures of concussion incidence were calculated. The overall suspected concussion rate was calculated using all reported concussions (logged + not-logged concussions) as the numerator and total player match hours as the denominator, expressed per 1000 player match hours. Athletic Exposures (AE) were also calculated to provide an incidence rate inclusive of both match and training exposures, with each training session and match equating to one exposure per player, expressed per 1000 AE. The alpha was set a priori at 0.05. Two outcomes were modelled: (1) ideal pathway adherence (ideal vs. non-ideal) and (2) SDP adequacy (adequate vs. too-short), analysed in separate models. For each outcome, two parallel modelling approaches were used: mixed-effects logistic regression (GLMM; lme4) as the primary model, and generalised estimating equations (GEE; geepack) with exchangeable correlation structure and cluster-robust standard errors as a sensitivity comparison. Where both approaches yielded consistent results, findings were considered robust. Predictors examined were ethnicity, physiotherapist presence, baseline completion, sex, age, grade, season, and provincial union, informed by prior literature. Provincial unions were treated as fixed covariates (n = 4), with Union 3 as the reference category. Club-level clustering was modelled via a random intercept (ICC = 10.4%), and player identity was included as a random intercept to account for repeat concussions (ICC = 0%; retained for methodological completeness). Union 4 was excluded from the SDP model due to complete separation. Missing data for the SDP outcome (28.2%) arose by design — players who did not return to play that season cannot be classified as adequate or too-short — and were excluded from that model; all other predictors were complete. Stratified analyses by grade, sex, and provincial union were conducted, and interaction terms (physiotherapist∗grade, baseline∗grade, physiotherapist∗sex) were tested via likelihood ratio tests. A sensitivity analysis restricted to first concussions only (n = 618) was also conducted.

## 3 Results

Over the study a total of 618 players reported a total of 655 suspected concussions (n = 553 males, n = 102 females; age mean ± SD = 19.8 ± 4.5 years, range 8–40), of which 631 (96%) were logged in the App, 8 concussions were not logged but healthcare touchpoint data was captured. A further 16 suspected concussions were reported at end of season follow up, these are included in the overall concussion incidence rates but excluded from all pathway analyses as no healthcare touchpoint data was obtained (n = 639 retained for analysis). For a detailed breakdown of the healthcare touchpoints see Supplementary Fig. 1. Twenty players sustained two suspected concussion and one sustained three. From the total number of reported suspected concussions (n = 639), 526 (82.3%) (System GP n = 408; Own GP n = 118) were diagnosed (n = 448 males, n = 78 females). Demographic details of participants are reported in Table 1. The overall suspected concussion rate across the three seasons (n = 655) was 16.6/1000 player match hrs (2020 = 13.6, 2021 = 18.5, 2022 = 17.2/1000 player match hrs). The suspected concussion injury rates for matches and trainings combined were 4.3/1000 AE (2020 = 4.7, 2021 = 5.7, 2022 = 3.0/1000 AE). The overall diagnosed concussion rate was 12.1/1000 player match hrs (2020 = 11.4, 2021 = 12.3, 2022 = 11.8/1000 player match hrs) and 2.7/1000 AE (match and training) (2020 = 3.5, 2021 = 4.0, 2022 = 1.8/1000 AE).

**Table 1:** Demographic details and healthcare touchpoints for players who sustained a suspected concussion (SC) during the 2020–22 seasons (N = 639).

Demographic variable	2020 n = 125	2021 n = 258	2022 n = 256	Total N = 639
**Sample who reported a suspected concussion**				
Age (Mean ± SD)	19.8 ± 4.5	19.7 ± 4.1	20.1 ± 5.1	19.9 ± 4.6
**Sex**				
Male	111 (88.8%)	219 (84.9%)	220 (85.9%)	550 (86.1%)
Female	14 (11.2%)	39 (15.1%)	36 (14.1%)	89 (13.9%)
**Grade**				
Premier	84 (67.2%)	161 (62.4%)	155 (60.5%)	400 (62.6%)
High school	41 (32.8%)	93 (36.0%)	95 (37.1%)	229 (35.8%)
Junior	0	4 (1.6%)	6 (2.3%)	10 (1.6%)
**Ethnicity**				
NZ European	70 (56.0%)	141 (54.7%)	139 (54.3%)	350 (54.8%)
Māori	28 (22.4%)	62 (24.0%)	54 (21.1%)	144 (22.5%)
Pasifika/Other	27 (21.6%)	55 (21.3%)	63 (24.6%)	145 (22.7%)
**Concussion management variables**				
Total logged SCs (% of reported)	124 (99.2%)	255 (99.2%)	252 (98.4%)	631 (98.7%)
Baseline test completed	103 (82.4%)	187 (72.5%)	160 (62.5%)	450 (70.4%)
Team had physio support	90 (72.0%)	171 (66.3%)	167 (65.2%)	428 (67.0%)
Attended ED	2 (1.6%)	12 (4.7%)	22 (8.6%)	36 (5.6%)
**GP Diagnosis^a^**				
System GP	77 (61.6%)	175 (67.8%)	156 (60.9%)	408 (63.8%)
Own GP	17 (13.6%)	37 (14.3%)	64 (25.0%)	118 (18.5%)
None	31 (24.8%)	46 (17.8%)	36 (14.1%)	113 (17.7%)
**Medical Clearance^b^**				
System GP	80 (64.0%)	136 (52.7%)	144 (56.2%)	360 (56.3%)
Own GP	4 (3.2%)	15 (5.8%)	27 (10.5%)	46 (7.2%)
Not required	8 (6.4%)	21 (8.1%)	28 (10.9%)	57 (8.9%)
None	33 (26.4%)	86 (33.3%)	57 (22.3%)	176 (27.5%)
**Stand-down period**				
Appropriate	75 (60.0%)	140 (54.3%)	133 (52.0%)	348 (54.5%)
Too short	12 (9.6%)	19 (7.4%)	23 (9.0%)	54 (8.5%)
No RTP during respective season^c^	30 (24.0%)	78 (30.2%)	72 (28.1%)	180 (28.2%)
Not concussed at diagnosis	8 (8.0%)	21 (8.1%)	28 (11.3%)	57 (8.9%)

**Ideal pathway followed**	60 (48.0%)	127 (49.2%)	132 (51.6%)	319 (49.9%)

SC = suspected concussion. Ideal pathway: logged concussion + GP diagnosis (system or own) + GP medical clearance (system, own, or not required). ˆa GP Diagnosis: 'System GP' = CMP-affiliated GP who used the NZR Portal; 'Own GP' = player's regular GP (outcome captured by research assistant follow-up); 'None' = no diagnosis visit documented.

ˆb Medical Clearance: 'Not required' = player cleared at diagnosis visit (not concussed); 'None' = no clearance visit documented.

ˆc No RTP includes: retired due to symptoms/precaution (n = 39), missing data (n = 34), retired/extended stand-down due to end of season or COVID disruption (n = 89), referred to ACC/concussion services (n = 18) — see SDP code breakdown.

Following a suspected concussion, the ideal management pathway was adhered to by 319 players (50%). The remaining 320 missed at least one step in the ideal pathway. Ideal pathway adherence was consistent across seasons. A breakdown in adherence is shown in Fig. 3 and details of CMP steps in Table 1.

**Fig. 3:**
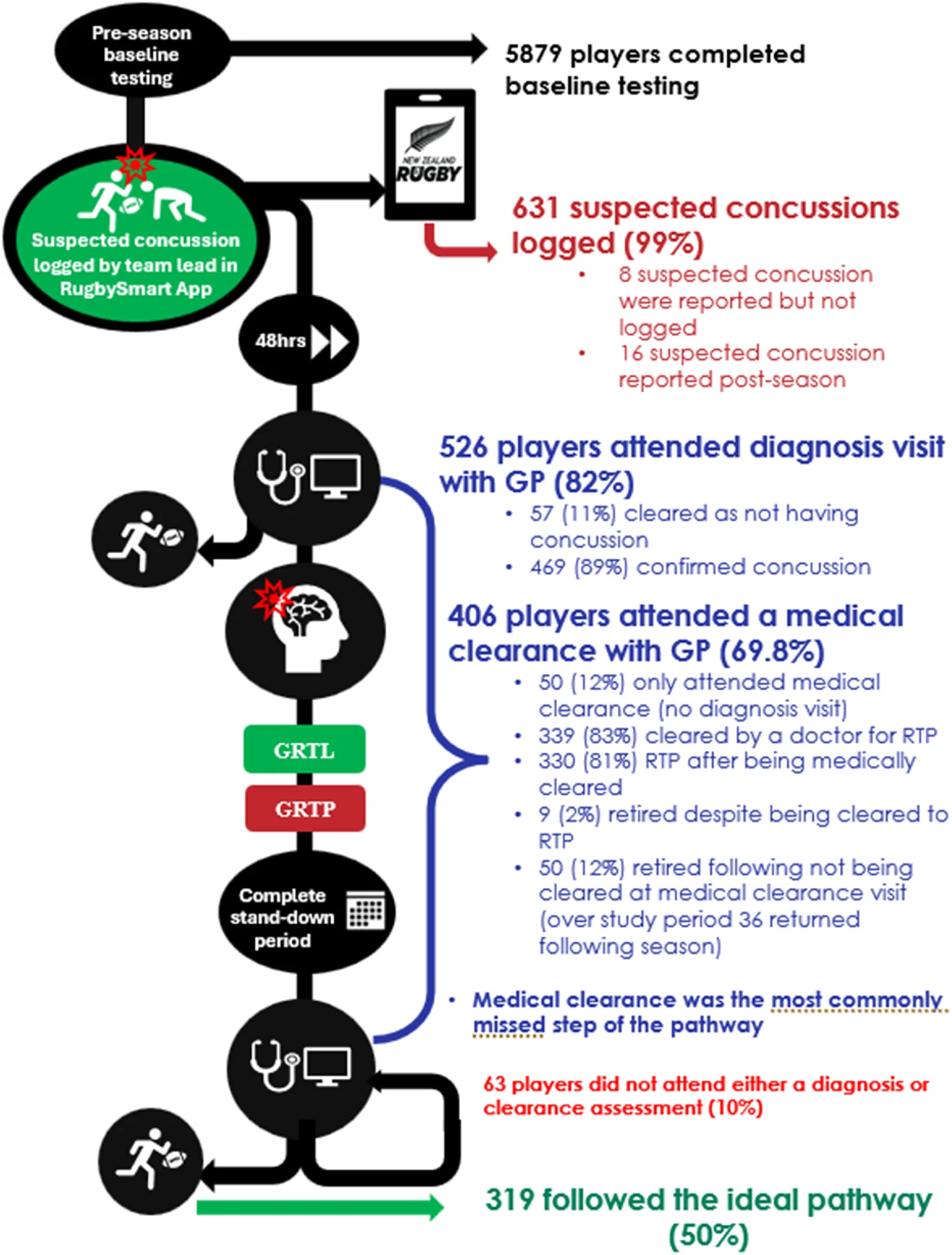
Breakdown of the compliance to each step in the recommended pathway for the NZR CMP.

Analysis of the GLMM and GEE results for ideal pathway adherence were consistent across both modelling approaches (see Table 2; full GLMM/GEE comparison in Supplementary Table S2). Players who had completed a baseline assessment had 90% higher odds of following the ideal pathway (OR = 1.90, 95% CI: 1.26–2.85, *p* ≤ 0.01). The presence of a team physiotherapist was associated with higher odds of ideal pathway adherence (OR = 1.46, 95% CI: 0.84–2.53, *p* = 0.18), though this did not reach statistical significance in the primary model. Stratified analyses revealed a significant effect by Provincial Union where players in Provincial Union 3 had 6.15 times (95% CI: 2.12–17.9, *p* = 0.001) higher odds of following the ideal pathway if they had a team physiotherapist, suggesting physiotherapists play a particularly critical role in regions without dedicated clinic infrastructure. Players in the Provincial Union with access to a dedicated concussion clinic (Provincial Union 1) had significantly higher odds of ideal pathway adherence compared to Provincial Unions where players accessed GPs through the primary care setting (Union 3; OR = 2.67, 95% CI: 1.47–4.87, *p* = 0.001). Both Provincial Unions 2 and 3 did not differ significantly (OR = 0.76, p = 0.54), confirming both represent the standard primary care model. Provincial Union 4, who was in the first year of the study in 2022, showed substantially lower adherence (OR = 0.21, 95% CI: 0.05–0.84, *p* = 0.03; n = 32) Pasifika/Other players had 40% lower odds of adhering to the ideal pathway when compared to NZ European players (OR = 0.60, 95% CI: 0.38–0.94, *p* = 0.02). Stratified analyses indicated this effect was concentrated in premier grade players (OR = 0.40, 95% CI: 0.23–0.70, *p* = 0.001) and was not present in high school players (OR = 1.42, 95% CI: 0.64–3.18, *p* = 0.39). Adherence by Māori players did not differ significantly from NZ European players (OR = 1.00, 95% CI: 0.65–1.56, *p* = 0.98). Playing grade, sex, and age were not significantly associated with ideal pathway adherence in the primary model. Season 2022 showed higher odds compared to 2020 in the GLMM (OR = 1.86, 95% CI: 1.14–3.03, *p* = 0.01). All interaction terms tested (physio∗grade, baseline∗grade, physio∗sex) were non-significant (all *p* ≥ 0.08). Stratified analyses by grade, sex, and provincial union are presented in Supplementary Table S3. A sensitivity analysis restricted to first concussions only (n = 618) produced results consistent with the primary model, with baseline completion remaining the strongest predictor (OR = 1.99, 95% CI: 1.32–3.01, *p* = 0.001) and no meaningful change in direction or magnitude for other predictors, supporting the robustness of the primary findings.

**Table 2:** Mixed-effects logistic regression (GLMM) results for the odds of following the ideal pathway through the CMP (N = 639). GEE sensitivity analysis results are consistent with these findings and are presented in Supplementary Table S1.

Variable	OR	95% CI	*p*-value
**Baseline assessment (ref: Not completed)**			
Completed	1.90^a^	1.26–2.85	0.002
**Team physiotherapist (ref: No)**			
Yes	1.46	0.84–2.53	0.18
**Ethnicity (ref: NZ European)**			
Māori	1.00	0.65–1.56	0.98
Pasifika/Other	0.60^a^	0.38–0.94	0.02
**Playing grade (ref: Premier)**			
High school	1.73	0.90–3.32	0.10
**Sex (ref: Male)**			
Female	0.86	0.46–1.61	0.64
Age (per year, centred)	1.03	0.98–1.09	0.23
**Season (ref: 2020)**			
2021	1.21	0.76–1.93	0.42
2022	1.86^a^	1.14–3.03	0.01
**Provincial union (ref: Union 3)^a^**			
Union 1	2.67^a^	1.47–4.87	0.001
Union 2	0.76	0.32–1.82	0.54
Union 4	0.21^a^	0.05–0.84	0.03

*Club-level ICC*^*b*^*= 10.4%; player-level ICC = 0.0%*			

OR = odds ratio; CI = confidence interval. Reference categories: NZ European (ethnicity), Male (sex), Premier (grade), Season 2020, Baseline not completed, No physio, Union 3 (provincial union). Union 3 = primary care GP network (reference); Union 1 = dedicated concussion clinic; Union 2 = primary care GP network; Union 4 = primary care GP network, small n (n = 32), interpret with caution.

ˆa Provincial union treated as fixed covariate (four units; insufficient for random-effects variance estimation).

ˆb Club-level ICC = 10.4%, confirming meaningful within-club clustering; player-level ICC = 0.0%.

a Denotes statistical significance at an alpha = 0.05.

### 3.1 Following the correct stand-down period

Of the 639 suspected concussions where healthcare touchpoint data were captured, 348 (54.5%) completed an adequate SDP, 54 (8.5%) returned too early, 57 (8.9%) were not concussed at diagnosis, 39 (6.1%) retired due to symptoms/precaution, 34 (5.3%) had missing data, 88 (13.8%) retired or had extended SDP due to end of season or COVID disruption, and 19 (3.0%) were referred to ACC concussion services (See Supplementary Fig. 2). Among players who followed the ideal pathway (n = 319), 85.0% (n = 271) RTP during that season, which is significantly higher when compared to those who did not follow the ideal pathway (n = 320), where only 57.8% (n = 185) RTP.

A GLMM was used to explore SDP adherence, comparing players who completed an appropriate SDP vs those who returned too early. Union 4 was excluded from the model due to complete separation – no players in this union returned to play prematurely – leaving a model sample of n = 439 (appropriate = 379; too short = 60). The model was non-significant across all predictors (see Table 3; GEE sensitivity analysis in Supplementary Table S3). Baseline completion showed a borderline trend toward adequate SDP (OR = 1.75, 95% CI: 0.93–3.30, *p* = 0.08), suggesting a possible protective effect that may be underpowered in this sample. Stratified analyses are presented in Supplementary Table S4.

**Table 3:** Mixed-effects logistic regression (GLMM) results for the odds of completing an adequate stand-down period versus returning too early (binary outcome; n = 439). GEE sensitivity analysis results are presented in Supplementary Table S2.

Variable	OR	95% CI	*p*-value
**Baseline assessment (ref: Not completed)**			
Completed	1.75	0.93–3.30	0.08
**Team physiotherapist (ref: No)**			
Yes	1.34	0.59–3.04	0.48
**Ethnicity (ref: NZ European)**			
Māori	1.41	0.66–3.03	0.38
Pasifika/Other	1.85	0.84–4.06	0.13
**Playing grade (ref: Premier)**			
High school	0.62	0.27–1.41	0.25
**Sex (ref: Male)**			
Female	0.87	0.34–2.23	0.77
Age (per year, centred)	1.00	0.91–1.11	0.94
**Season (ref: 2020)**			
2021	1.57	0.75–3.26	0.23
2022	1.36	0.65–2.84	0.42
**Provincial union (ref: Union 3)^a^**			
Union 1	0.94	0.44–2.02	0.88
Union 2	0.53	0.22–1.29	0.16

n = 439 (Union 4 excluded — complete separation: no players in Union 4 returned to play prematurely). Outcome: 1 = Adequate SDP (ref: 0 = Too short). No predictors reached statistical significance. GEE sensitivity analysis results in Supplementary Table S2.

OR = odds ratio; CI = confidence interval. Outcome: adequate stand-down period (adequate SDP and players not diagnosed with concussion) vs. too-short SDP. Players who did not return to play (retired, end of season, COVID disruption, missing, referred to ACC concussion services) were excluded from this model (n = 200 excluded; n = 439 retained).

ˆa Union 4 excluded due to complete separation (no players in Union 4 returned too early).

## 4 Discussion

The presence of concussion management guidelines represents an important first step toward player safety; however, guidelines alone have proven insufficient to drive meaningful behavioural change [9]. As the governing body for rugby in NZ, NZR sought to explore how technology could support four key concussion management priorities: (1) recognition of symptoms, (2) removal from play, (3) recovery through medical diagnosis, and (4) return via medical clearance after completion of the SDP. Across three seasons, 96% of suspected concussion were logged in the NZR App—a substantial improvement from 70% in 2019 [15]. Half of all players completed the ideal pathway, compared with just 28% in 2019. Players who completed the ideal pathway were also more likely to adhere to the prescribed SDP, thereby reducing the risk of premature RTP. Our analysis identified that completion of a pre-season baseline assessment increased the odds by 90% that a player would follow the ideal pathway.

Capturing injury data and healthcare touchpoints in community sport remains challenging due to reliance on self-reporting and variable access to medical personnel. We need to develop strategies and user-friendly tools that create supportive environments that encourage and assist medical and sports personnel in their guideline implementation efforts. This study's large and diverse cohort (n = 5879) encompassing both sexes, various playing grades and teams from both urban and rural centres, highlights that a technology-enabled process can overcome several logistical barriers to reporting and follow-up.

Research across community sport has documented high levels of under-reporting of suspected concussions, which has a significant impact on the recognition of concussion [[6], [7], [8]]. In the current study, the concussion incidence observed (16.6 per 1000 player match-hours) exceeded rates previously reported in NZ community rugby (12.37/1000) [15] and other amateur cohorts [[16], [17], [18], [19]]. While this could reflect a true increase in concussions, it more likely represents improved recognition and reporting through the structured CMP [20,21]. The NZR App likely enhanced clarity and accountability by identifying a team lead responsible for logging suspected concussion, contributing to high adherence (96%) and mirroring evidence that technology can effectively support concussion management [22]. Importantly, digital systems must extend beyond reporting to ensure clinical follow-through. In this sense, the App & GP Portal supported tracking of both diagnosis and clearance. Earlier studies as part of the broader project showed that physiotherapists found the App helpful for streamlining their workflow and appreciated having important information—like red flags—readily accessible. This increased their confidence in making decisions and gave them the authority to manage concussions appropriately [4,5]. However, due to the lack of baseline injury surveillance data and the presence of a control there may be other confounding factors such as greater concussion awareness from the media and national education campaigns that may have also contributed to improve identification over this time period.

In this study, 82% of players attended a diagnosis visit with either a GP who used the GP Portal or their own doctor, an improvement from the 76% in 2019 [15]. Similarly, adherence to medical clearance increased to 70%, compared to 64% in 2019 [15]. Of interest is the consistent pattern observed in both the current study and 2019 where attendance to a medical clearance is lower than the diagnosis assessment. Players were interviewed previously about their treatment experience post-concussion [15]. They highlighted that attending a diagnosis and clearance visit was facilitated by easy and timely access to GPs, supported mainly through receiving clear instructions by their team lead/physiotherapist, or a referral to the GP [15]. In a study exploring factors associated with concussion management in female Gaelic Football players, receiving a concussion diagnosis increased the odds of a player following the RTP process and obtaining medical clearance [23]. World Rugby's community guidelines state that players should receive a medical clearance prior to RTP, yet in a study in community rugby in Australia 78% of players received no RTP advice [22]. Encouragingly, in the current study, 78% of players were medically cleared before RTP. The findings from the current study indicate that improving adherence with the medical clearance stage in the CMP should be a focus, the NZR App appears to support the increased recognition and reporting of a concussion and that engagement in the process increases the likelihood of a player attending both a diagnosis and medical clearance [24,25].

Overall, 50% of players adhered to the ideal pathway—a notable improvement from 28% in 2019 [15]. Ideal pathway adherence carried meaningful clinical consequences beyond compliance: players who completed the ideal pathway were more likely to RTP that season (85%) compared to those who did not (57.8%). This finding reinforces that the value of pathway adherence extends beyond regulatory compliance to tangible player outcomes. In a study examining adherence rates to a recommended pathway in primary care clinics in a Children Hospital Network, 80% of patients met the adherence requirements [1]. The difference in adherence rates between the current study and this may be explained by the difference in point of entry into the CMP. The initial point of contact in the current study was pitch-side where suspected concussions were identified and logged, whereas the initial contact point for Mohammed et al. was when the patient contacted the clinic, which likely explains the higher adherence, as they are pro-actively seeking care. Previous research has highlighted that non-adherence to the recommended guidelines for a concussion is associated with poorer outcomes [1,23]. Taken together these and previous findings highlight that timely and proper evaluation and treatment of a concussion mitigate the risk of protracted recovery, emphasizing the critical need for access to appropriate care and adherence to care recommendations [1,23].

To encourage future adherence to the ideal pathway, it was essential to understand what key facilitators improved pathway adherence. The presence of a team physiotherapist was associated with higher odds of ideal pathway adherence in the primary model (OR = 1.46), though this did not reach statistical significance. Literature confirms the vital role of a physiotherapist in the management of concussion [5,24,26,27]. Interviews with players have highlighted physiotherapists as playing an influential role in their concussion management journey; through initial recognition; to instructing the player to go and see a GP, and in providing guidance and support during their recovery [24,27]. A trusting physiotherapist–player relationship provides an opportunity to positively influence players' adherence [23]. Stratified analyses revealed that the significance of this effect varied considerably by union: in the union without access to a dedicated concussion clinic, the presence of a team physiotherapist was associated with substantially higher odds of ideal pathway adherence (OR = 6.15, *p* = 0.001). This suggests that physiotherapists may serve a critical compensatory role in settings where formal clinic infrastructure is absent, effectively bridging the gap between pitch-side recognition and clinical follow-up. However, access to care and the scarcity of qualified healthcare professionals associated with teams at this level, continues to pose a challenge to implementing community concussion guidelines [4,5,26,28,29].

Complementing the role of personnel, access to structured clinical infrastructure also emerged as a significant determinant of adherence. Players in the union with access to a dedicated primary care concussion clinic had significantly higher odds of ideal pathway adherence compared to those accessing GPs through standard primary care (OR = 2.67). This finding highlights that clinic infrastructure plays an independent role in supporting pathway completion, and has implications for how governing bodies allocate resources across regions. Taken together, the physiotherapist and access to a primary care concussion clinic findings suggest a tiered model of support: where dedicated clinical infrastructure exists, adherence is facilitated; where it does not, physiotherapist presence may partially compensate. Strategies to find sustainable models for subsidised, localised healthcare access at the grassroots level should be prioritised.

The second factor that improved player adherence was completing a pre-season baseline assessment, where a player with a baseline assessment had 1.90 times higher odds of following the ideal pathway. The value of baseline testing has previously been described to extend beyond its role in clinical assessment [25,30]. The use of a baseline assessment at the start of the season may have been a key factor that engaged the player in the CMP process, by providing an interactive educational opportunity that included information about symptoms and functional deficits [4,25]. Engagement in the process may have brought the seriousness of concussions to the forefront for players. Importantly, baseline testing engages players early in the process and ensures they are aware of the recommended processes from the start [4,25].

Research examining health disparities and inequities in access to healthcare experienced by different ethnic and sociodemographic groups is driven by structural system-level factors that can negatively impact an individual's experience of care [1]. These drivers are often multifactorial in nature and intersect (systemic racism and poverty), which then proceed to influence socioeconomic outcomes that lead to health disparities [1]. Numerous studies have found that Black, Hispanic, individuals with limited English proficiency and those with public insurance are less likely to access care and follow the recommended guidelines [1,31]. In the current study players who self-identified as Pasifika had 40% lower odds of pathway adherence compared to NZ Europeans (OR = 0.60). This finding is concerning and suggest that culturally responsive strategies are needed to facilitate adherence.

Previous research indicate when no structured CMP is in place, non-adherence with SDP requirements can be high [22]. In this study, adherence to the recommended SDP was achieved by 62% of players, with 8% RTP prematurely. This represents an improvement compared from previous NZ figures (52% adherence). [15]. However, the slight increase in the proportion of players returning to play too early, from 7% in the previous study [15] to 8% in the current study, indicates that some challenges with adherence still remain. The ongoing challenge remains consistent buy-in from all stakeholders, including players [6,32]. Qualitative findings from earlier work suggested that ‘belief in the value of pathway and diligence in its execution’ were critical factors for adherence [15]. Strategies that facilitate buy-in and positive attitudes towards CMP should remain a priority.

The large sample size, including both males and females, from a range of geographical and socioeconomic areas, allows generalizability of the results to the community rugby setting in NZ. The prospective nature allowed us to track suspected concussions in real time, and to identify potential barriers and facilitators to the process. Nonetheless, some limitations exist. Training exposure was captured post-season with teams providing training schedules, which may be subject to recall and measurement bias. During the 2020 season community rugby was impacted by COVID-19, which resulted in pauses in the season based on government policy, schedule changes, reduced player availability, and altered healthcare access. Thus ‘season’ was included as a fixed covariate in all models to adjust statistically for year-on-year variation in outcomes. In addition, the study employed a prospective cohort design without a concurrent control group, and comparisons with pre-intervention data should be interpreted with caution. Observed improvements over time may reflect the effect of the CMP and App, but contributions from secular trends in concussion awareness, broader policy changes, or pandemic-era shifts in healthcare-seeking behaviour cannot be excluded. Furthermore, union-level differences in CMP implementation — specifically the availability of a dedicated concussion clinic in one union — were not captured as an individual-level variable, meaning the union effects reported here represent a composite of geographic, structural, and resource-related factors. Future research using a controlled or stepped-wedge design, with individual-level data on healthcare access, would strengthen causal inference regarding the effectiveness of technology-enabled concussion management pathways.

Collectively, these findings demonstrate that a multi-component, technology-enabled intervention improved adherence to CMP in community rugby. These results carry practical and policy implications for governing bodies and grassroots organizations. Practically, the integration of baseline testing and dedicated medical staff, particularly physiotherapists into team structures may facilitate adherence to concussion guidelines, with the greatest gains observed where physiotherapists operate in the absence of formal clinic infrastructure. Where clinical infrastructure can be established, its impact on adherence is substantial and should be pursued as a longer-term structural goal. From a policy perspective, bridging the resource gap remains a challenge. Strategies to find sustainable models for subsidized, localized healthcare access at the grassroots level should be prioritized. Healthcare support is necessary to ensure that the initial *Recognise and Remove* step, facilitated by the use of technology, is consistently followed by clinical diagnosis and safe clearance. Ultimately, combining technological innovation with culturally responsive, relationship-driven strategies offers the best path toward equitable and effective concussion management in community sport.

## 5 Conclusion

Findings from this study indicate improvement in adherence to the ideal CMP with the use of technology. Completing a pre-season baseline assessment was the strongest and most consistent predictor of adherence. Access to dedicated clinical support, through an acute care concussion clinic, primary care GP or a team physiotherapist, was also associated with higher levels of pathway completion. Players who completed the ideal pathway were substantially more likely to return to play that season, underscoring the clinical value of full pathway adherence. Equity gaps remain a concern, particularly for Pasifika players, highlighting that technology alone is insufficient to address structural and cultural barriers to care. The positive uptake across diverse player groups provides a strong foundation for future improvements. Enhancing outcomes for Pasifika players will require leveraging community strengths, partnerships, and culturally grounded approaches alongside the technology. Efforts to expand education, improve access to healthcare, and strengthen policy support may help ensure that all players are equally able to complete the SDP and obtain medical clearance before returning to play. Future work should investigate other factors that influence players’ engagement with concussion protocols. Integrating technology with ongoing education and medical support appears to be a promising strategy to promote safe and consistent concussion management in community rugby.

## Ethics declaration

Written informed consent to take part in the study and to publish the article has been obtained from all participants or their legal representatives. The privacy rights of participants have been observed.

This study was performed in compliance with relevant laws, regulatory frameworks and guidelines where the research took place. This study was approved by the University of Otago (Approval No. 18/087).

## Ethics approval and consent to participate

Approval to conduct the research was obtained from the University of Otago Human Ethics Committee (approval 18/087) to conduct this project.

## Consent for publication

N/a.

## Availability of data and materials

The datasets used and/or analysed during the current study are available from the corresponding author on reasonable request.

## Funding

This work formed part of NZR's National Concussion Strategy and was funded by World Rugby and the Accident Compensation Corporation (ACC) through the RugbySmart programme. The funders did not direct data collection, analysis, interpretation, manuscript preparation, or the decision to submit for publication.

## Declaration of competing interest

The authors declare the following financial interests/personal relationships which may be considered as potential competing interests:

Dr Danielle Salmon reports financial support was provided by World Rugby Limited. Dr Danielle Salmon reports financial support was provided by New Zealand Rugby Foundation. Dr Danielle Salmon reports financial support was provided by Accident Compensation Corp. Dr Danielle Salmon reports a relationship with New Zealand Rugby that includes: employment. Janelle Romanchuk, Marelise Badenhorst, Anja Zoellner were employed or contracted by New Zealand Rugby to support this research. If there are other authors, they declare that they have no known competing financial interests or personal relationships that could have appeared to influence the work reported in this paper.
