# Supplementary material for: Predictors of concussion management pathway adherence in New Zealand community rugby: A prospective cohort study
Source: JSAMS Plus. 2026 Oct 5;8:100161. doi: 10.1016/j.jsampl.2026.100161 (PMC13672860; doi:10.1016/j.jsampl.2026.100161)
Supplement: Supplementary file 1 [file mmc1.docx]

**Supplementary Table S1.** STROBE Statement—checklist of items that should be included in reports of observational studies

|  | Item No. | Recommendation | Page  No. | Relevant text from manuscript |
| --- | --- | --- | --- | --- |
| **Title and abstract** | 1 | (*a*) Indicate the study’s design with a commonly used term in the title or the abstract | Abstract and Page 4 (methods) | This study employed a prospective observational cohort study to examine concussion management adherence across four provincial unions (PU) in New Zealand (NZ). |
|  |  | (*b*) Provide in the abstract an informative and balanced summary of what was done and what was found | Methods and Results section of Abstract | Refer to Abstract |
| Introduction | | | |  |
| Background/rationale | 2 | Explain the scientific background and rationale for the investigation being reported | Page 4 Introduction and Study design (Methods) | Refer to Page 4 |
| Objectives | 3 | State specific objectives, including any prespecified hypotheses |  | The aim of this study was to evaluate adherence to an ideal concussion management pathway, facilitated through the use of an NZR App and GP Portal. To explore what factors contributed to improved adherence to the ideal pathway the effect of gender, ethnicity, grade, presence of team physiotherapist and completion of a baseline assessment were examined. In addition to pathway adherence, compliance with the appropriate SDP was examined to determine alignment with NZR guidelines in effect at the time of the study (21 days for players aged ≥19 years and 23 days for players aged <19 years) and to identify those who returned early |
| Methods | | | |  |
| Study design | 4 | Present key elements of study design early in the paper | Page 4 (Methods) | Refer to page 4 |
| Setting | 5 | Describe the setting, locations, and relevant dates, including periods of recruitment, exposure, follow-up, and data collection | Page 4 and 5 (Methods) | Refer to page 4 and 5 |
| Participants | 6 | (*a*) *Cohort study*—Give the eligibility criteria, and the sources and methods of selection of participants. Describe methods of follow-up  *Case-control study*—Give the eligibility criteria, and the sources and methods of case ascertainment and control selection. Give the rationale for the choice of cases and controls  *Cross-sectional study*—Give the eligibility criteria, and the sources and methods of selection of participants | Page 6 (Participants) | All registered players in consenting high school and club teams were eligible to participate. |
| Variables | 7 | Clearly define all outcomes, exposures, predictors, potential confounders, and effect modifiers. Give diagnostic criteria, if applicable | Page 6-7 (Data processing and analysis) | Refer to page 6-7 |
| Data sources/ measurement | 8* | For each variable of interest, give sources of data and details of methods of assessment (measurement). Describe comparability of assessment methods if there is more than one group | Page 6-7 (Data processing and analysis) | Refer to page 6-7 |
| Bias | 9 | Describe any efforts to address potential sources of bias | Page 14 - bias discussed in limitations |  |
| Study size | 10 | Explain how the study size was arrived at | Page 6 (Participants) | All registered players in consenting high school and club teams were eligible to participate. |

| Quantitative variables | 11 | Explain how quantitative variables were handled in the analyses. If applicable, describe which groupings were chosen and why | Page 6-7 (Data processing and analysis) | Predictors examined were ethnicity, physiotherapist presence, baseline completion, sex, age, grade, season, and provincial union, informed by prior literature.[42] Provincial unions were treated as fixed covariates (n=4), with Union 3 as the reference category. Club-level clustering was modelled via a random intercept (ICC=10.4%), and player identity was included as a random intercept to account for repeat concussions (ICC=0%; retained for methodological completeness). Union 4 was excluded from the SDP model due to complete separation. Missing data for the SDP outcome (28.2%) arose by design — players who did not return to play that season cannot be classified as adequate or too-short — and were excluded from that model; all other predictors were complete. |
| --- | --- | --- | --- | --- |
| Statistical methods | 12 | (*a*) Describe all statistical methods, including those used to control for confounding | Page 6-7 (Data processing and analysis) | Data were analysed using R (Version 4.5). Descriptive statistics were presented for demographics, baseline completion, presence of a team physiotherapist, RTP outcomes, and SDP adherence. The alpha was set a priori at 0.05. Two outcomes were modelled: (1) ideal pathway adherence (binary: ideal vs. non-ideal) and (2) SDP adequacy (binary: adequate vs. too-short), analysed in separate models. For each outcome, two parallel modelling approaches were used: mixed-effects logistic regression (GLMM; lme4) as the primary model, and generalised estimating equations (GEE; geepack) with exchangeable correlation structure and cluster-robust standard errors as a sensitivity comparison. Where both approaches yielded consistent results, findings were considered robust.  Stratified analyses by grade, sex, and provincial union were conducted, and interaction terms (physiotherapist*grade, baseline*grade, physiotherapist*sex) were tested via likelihood ratio tests. A sensitivity analysis restricted to first concussions only (n=618) was also conducted. |
|  |  | (*b*) Describe any methods used to examine subgroups and interactions | Page 6-7 (Data processing and analysis) | Refer to page 6-7 |
|  |  | (*c*) Explain how missing data were addressed | Page 10 (Results) | Over the three seasons a total of 618 players reported a total of 655 suspected concussions (n= 553 males, n= 102 females; age mean ± SD = 19.8 ± 4.5 years, range 8-40), of which 631 (96.0%) were logged in the App, 8 concussions were not logged but healthcare touchpoint data was captured, as the research team was notified when these happened. A further 16 suspected concussions were reported at end of season follow up, these are included in the overall concussion incidence rates but excluded from all pathway analyses as no healthcare touchpoint data was obtained (n=639 retained for analysis).For a detailed breakdown of the healthcare touchpoints see **Supplementary Figure 1**.  Union 4 was excluded from the binary SDP model due to complete separation – no players in that union returned to play prematurely – leaving a model sample of n=439 (appropriate=379; too short=60). |
|  |  | (*d*) *Cohort study*—If applicable, explain how loss to follow-up was addressed | Page 10 Results | Table 1 provides a summary of what data was included in the analysis and what data was missing. |
|  |  | (*e*) Describe any sensitivity analyses | Page 7 (data analysis) | A sensitivity analysis restricted to first concussions only (n=618) was also conducted. |
| Results | | | | |
| Participants | 13* | (a) Report numbers of individuals at each stage of study—eg numbers potentially eligible, examined for eligibility, confirmed eligible, included in the study, completing follow-up, and analysed | Table 1 and Supplementary Files 1 and 2 |  |
|  |  | (b) Give reasons for non-participation at each stage | Table 1 | Table 1 provides the details around SDP adherence, however no further information was captured for reason for non-compliance to the ideal pathway. |
|  |  | (c) Consider use of a flow diagram | Figure 3 |  |
| Descriptive data | 14* | (a) Give characteristics of study participants (eg demographic, clinical, social) and information on exposures and potential confounders | Table 1 |  |
|  |  | (b) Indicate number of participants with missing data for each variable of interest | Supplementary Figures 1 and 2 |  |
|  |  | (c) *Cohort study*—Summarise follow-up time (eg, average and total amount) | N/A |  |
| Outcome data | 15* | *Cohort study*—Report numbers of outcome events or summary measures over time | Table 1, Figure 3 and Supplementary Figures 1 and 2 |  |
| Main results | 16 | (*a*) Give unadjusted estimates and, if applicable, confounder-adjusted estimates and their precision (eg, 95% confidence interval). Make clear which confounders were adjusted for and why they were included | Page 8 and 9 (Results) Table 2 and 3 |  |

| Other analyses | 17 | Report other analyses done—eg analyses of subgroups and interactions, and sensitivity analyses | Page 9 (Results) | A sensitivity analysis restricted to first concussions only (n=618) produced results consistent with the primary model, with baseline completion remaining the strongest predictor (OR=1.99, 95% CI: 1.32–3.01, p=0.001) and no meaningful change in direction or magnitude for other predictors, supporting the robustness of the primary findings. |
| --- | --- | --- | --- | --- |
| Discussion | | | | |
| Key results | 18 | Summarise key results with reference to study objectives | Page 10-14 (Discussion) | Refer to page 10 -14 |
| Limitations | 19 | Discuss limitations of the study, taking into account sources of potential bias or imprecision. Discuss both direction and magnitude of any potential bias | Page 14 | Team exposure data was captured post-season with teams providing training schedules and the number of pre-season matches, which may be subject to recall bias. Team managers acting as primary injury recorders may have been a limitation, given they do not have any medical training. However, the ability for a suspected concussion to be logged and then later confirmed by a medical professional allowed mitigation of this risk. This also allowed players to get medical input even where there was no medical personnel present at the match or training where the injury happened. During the 2020 season community rugby was impacted by COVID-19, which resulted in pauses in the season based on government policy, schedule changes, reduced player availability, and altered healthcare access. Thus ‘season’ was included as a fixed covariate in all models to adjust statistically for year-on-year variation in outcomes. In addition, the study employed a prospective cohort design without a concurrent control group, and comparisons with pre-intervention data should be interpreted with caution. Observed improvements over time may reflect the effect of the CMP and App, but contributions from secular trends in concussion awareness, broader policy changes, or pandemic-era shifts in healthcare-seeking behaviour cannot be excluded. Furthermore, union-level differences in CMP implementation — specifically the availability of a dedicated concussion clinic in one union — were not captured as an individual-level variable, meaning the union effects reported here represent a composite of geographic, structural, and resource-related factors. |
| Interpretation | 20 | Give a cautious overall interpretation of results considering objectives, limitations, multiplicity of analyses, results from similar studies, and other relevant evidence | Page 15 (Discussion) | Findings from this study indicate improvement in adherence to the ideal concussion management pathway with the use of technology. Pre-season baseline assessment was the strongest and most consistent predictor of adherence, and access to dedicated clinical infrastructure — whether through a concussion clinic or a team physiotherapist in settings without one — was associated with higher pathway completion. Players who completed the ideal pathway were substantially more likely to return to play that season, underscoring the clinical value of full pathway adherence. |
| Generalisability | 21 | Discuss the generalisability (external validity) of the study results | Page 14 | The large sample size, including male and female data, from a range of geographical and socioeconomic areas, allows generalizability of the results to the community rugby setting in NZ. |
| Other information | |  | | |
| Funding | 22 | Give the source of funding and the role of the funders for the present study and, if applicable, for the original study on which the present article is based | Title page | Funding for this study as part of NZR National Concussion Strategy was provided by World Rugby and ACC through the RugbySmart program. |

*Give information separately for cases and controls in case-control studies and, if applicable, for exposed and unexposed groups in cohort and cross-sectional studies.

**Note:** An Explanation and Elaboration article discusses each checklist item and gives methodological background and published examples of transparent reporting. The STROBE checklist is best used in conjunction with this article (freely available on the Web sites of PLoS Medicine at http://www.plosmedicine.org/, Annals of Internal Medicine at http://www.annals.org/, and Epidemiology at http://www.epidem.com/). Information on the STROBE Initiative is available at www.strobe-statement.org.

**Supplementary Table S2.** Comparison of GLMM and GEE results for ideal pathway adherence (Outcome 1, N=639).

| **Variable** | **GLMM (primary)** | | | **GEE (sensitivity)** | | |
| --- | --- | --- | --- | --- | --- | --- |
|  | **OR** | **95% CI** | **p** | **OR** | **95% CI** | **p** |
| Baseline completed | 1.90 | 1.26–2.85 | 0.002 | 1.80 | 1.17–2.78 | 0.01 |
| Physio (Yes) | 1.46 | 0.84–2.53 | 0.18 | 1.40 | 0.78–2.51 | 0.26 |
| Ethnicity: Māori | 1.00 | 0.65–1.56 | 0.98 | 1.00 | 0.58–1.73 | 0.99 |
| Ethnicity: Pasifika/Other | 0.60 | 0.38–0.94 | 0.02 | 0.62 | 0.38–1.01 | 0.05 |
| Grade: High school | 1.73 | 0.90–3.32 | 0.10 | 1.68 | 0.95–2.97 | 0.08 |
| Sex: Female | 0.86 | 0.46–1.61 | 0.64 | 0.87 | 0.49–1.55 | 0.64 |
| Age (centred) | 1.03 | 0.98–1.09 | 0.23 | 1.03 | 0.99–1.08 | 0.12 |
| Season 2021 | 1.21 | 0.76–1.93 | 0.42 | 1.20 | 0.64–2.27 | 0.57 |
| Season 2022 | 1.86 | 1.14–3.03 | 0.01 | 1.78 | 0.89–3.57 | 0.11 |
| Union 1 | 2.67 | 1.47–4.87 | 0.001 | 2.45 | 1.42–4.25 | 0.001 |
| Union 2 | 0.76 | 0.32–1.82 | 0.54 | 0.71 | 0.25–2.03 | 0.53 |
| Union 4 | 0.21 | 0.05–0.84 | 0.03 | 0.22 | 0.07–0.69 | 0.01 |
| *Results were consistent across both approaches. GEE used exchangeable correlation structure with cluster-robust standard errors. Reference: Union 3, NZ European, Male, Premier grade, Season 2020, Baseline not completed, No physio.* | | | | | | |

*GLMM = generalised linear mixed model (lme4); GEE = generalised estimating equations (geepack) with exchangeable correlation structure and cluster-robust standard errors. Both models include club as clustering unit. Reference categories as per Table 2.*

**Supplementary Table S3.** Comparison of GLMM and GEE results for stand-down period adequacy (Outcome 2; adequate vs. too-short; n=439).

| **Variable** | **GLMM (primary)** | | | **GEE (sensitivity)** | | |
| --- | --- | --- | --- | --- | --- | --- |
|  | **OR** | **95% CI** | **p** | **OR** | **95% CI** | **p** |
| Baseline completed | 1.75 | 0.93–3.30 | 0.08 | 1.81 | 0.90–3.62 | 0.09 |
| Physio (Yes) | 1.34 | 0.59–3.04 | 0.48 | 1.48 | 0.68–3.23 | 0.32 |
| Ethnicity: Māori | 1.41 | 0.66–3.03 | 0.38 | 1.40 | 0.61–3.20 | 0.43 |
| Ethnicity: Pasifika/Other | 1.85 | 0.84–4.06 | 0.13 | 1.82 | 0.76–4.37 | 0.18 |
| Grade: High school | 0.62 | 0.27–1.41 | 0.25 | 0.45 | 0.24–0.82 | 0.01 |
| Sex: Female | 0.87 | 0.34–2.23 | 0.77 | 0.92 | 0.34–2.52 | 0.88 |
| Age (centred) | 1.00 | 0.91–1.11 | 0.94 | 0.98 | 0.89–1.07 | 0.58 |
| Season 2021 | 1.57 | 0.75–3.26 | 0.23 | 1.94 | 0.86–4.34 | 0.11 |
| Season 2022 | 1.36 | 0.65–2.84 | 0.42 | 1.34 | 0.58–3.12 | 0.50 |
| Union 1 | 0.94 | 0.44–2.02 | 0.88 | 1.04 | 0.55–1.95 | 0.91 |
| Union 2 | 0.53 | 0.22–1.29 | 0.16 | 0.53 | 0.22–1.30 | 0.16 |
| *Outcome: 1=Adequate SDP (ref: 0=Too short). Union 4 excluded (complete separation). n=44. GEE grade finding (OR=0.45, p=0.01) diverges from GLMM (OR=0.62, p=0.25) — interpret with caution. Reference categories: Union 3, NZ European, Male, Premier, Season 2020, Baseline not completed, No physio.* | | | | | | |

*GLMM = generalised linear mixed model (lme4); GEE = generalised estimating equations (geepack). Neither model identified significant predictors overall. The GEE grade finding (high school OR=0.45, p=0.01) was not replicated in the GLMM (OR=0.62, p=0.25) and should be interpreted with caution. Union 4 excluded (complete separation). Reference categories as per Table 3.*

**Supplementary Table S4.** Stratified GLMM results for ideal pathway adherence: selected strata (N=639).

| **Variable** | **Union 3 (n=145)** | **Union 1 (n=392)** | **Premier (n=400)** | **High school (n=229)** | **Male (n=550)** |
| --- | --- | --- | --- | --- | --- |
| Baseline completed | 2.09 (0.84–5.18) p=0.11 | 1.93 (1.17–3.18) p=0.01 | 2.49 (1.49–4.18) p=0.001 | 1.04 (0.50–2.17) p=0.92 | 2.19 (1.41–3.41) p<0.001 |
| Physio (Yes) | 6.15 (2.12–17.9) p=0.001 | 0.95 (0.42–2.17) p=0.91 | 1.21 (0.57–2.58) p=0.62 | 1.80 (0.71–4.53) p=0.22 | 1.44 (0.80–2.60) p=0.22 |
| Ethnicity: Pasifika/Other | 0.51 (0.17–1.51) p=0.22 | 0.51 (0.29–0.88) p=0.02 | 0.40 (0.23–0.70) p=0.001 | 1.42 (0.64–3.18) p=0.39 | 0.55 (0.34–0.89) p=0.02 |
| Ethnicity: Māori | 0.60 (0.25–1.44) p=0.25 | 0.71 (0.40–1.25) p=0.23 | 0.80 (0.46–1.38) p=0.41 | 1.74 (0.81–3.73) p=0.16 | 1.23 (0.76–2.00) p=0.41 |
| *ORs from GLMM (lme4), club-level random intercept. Union 2 (n=70) and Union 4 (n=32) strata not reported due to complete separation. Female stratum (n=89) underpowered; results not shown. All interaction terms non-significant (all LRT p≥0.08). Values shown as OR (95% CI) / p-value.* | | | | | |

*Results are shown for strata with adequate sample size for modelling. Within each stratum, all other model covariates are retained. Interaction terms physio × grade (LRT χ²=1.09, p=0.30), baseline × grade (χ²=2.97, p=0.08), and physio × sex (χ²=2.40, p=0.12) were all non-significant; stratified results are therefore descriptive.*

**Supplementary Figure F1.** C Concussion management pathway flow by pathway adherence outcome (ideal vs. alternative), across all concussions (n=639). MC = medical clearance.

**Supplementary Figure F2.** Concussion management pathway touchpoints and stand-down period (SDP) outcomes. MC = medical clearance. Note: Includes all 639 concussions; one provincial union (PU4) was excluded from the GLMM analysis due to complete separation.
